# The influence of CI electrode array design on the preservation of residual hearing

**DOI:** 10.3389/fneur.2025.1599369

**Published:** 2025-06-18

**Authors:** L. Fries, F. Everad, R. L. Beck, A. Aschendorff, S. Arndt, M. C. Ketterer

**Affiliations:** Department of Otorhinolaryngology, Medical Center – University of Freiburg, Faculty of Medicine, University of Freiburg, Freiburg, Germany

**Keywords:** electrode, residual hearing preservation, cochlear implant, speech perception, electrode array design

## Abstract

**Objectives:**

To individualize cochlear implant (CI) surgery regarding cochlear morphology, different electrode arrays have been developed. The study aims to investigate the influence of the electrode array design on the preservation of residual hearing, considering long-term results.

**Methods:**

We performed a retrospective analysis of 923 patients implanted with straight or perimodiolar electrode arrays from Cochlear™ and MED-EL between 2003 and 2021. The standard pure tone average (PTA4) and low-frequency PTA (PTAlow) were measured before and after surgery, with a follow-up period of 3.2 years up to 15.7 years.

**Results:**

In patients with measurable preoperative PTA4 (data of four frequencies), the slim straight electrode array (SSA) was chosen significantly more often preoperatively within the Cochlear™ portfolio (CA vs. SSA *p* = 0.007) and the Flex^24^ within the MED-EL portfolio (Flex^Soft^ vs. Flex^24^*p* = 0.0085). The electrode array design significantly influences the preservation of residual hearing, both in low-frequency PTA and standard PTA. The electrode arrays with the most favorable performance in terms of long-term residual hearing preservation appear to be the slim straight electrode array (SSA) from Cochlear™ and the Flex^24^ from MED-EL, with no statistical differences from other electrodes.

**Conclusion:**

Preoperative residual hearing influences the choice of electrode array within the manufacturer’s portfolio, with short electrode arrays showing superior results in the preservation of residual hearing. Over time, straight and short electrode arrays are associated with improved preservation of residual hearing. Therefore, for patients with existing relevant residual hearing, it is advisable to choose short and atraumatic lateral wall electrode arrays.

## Introduction

For cochlear implant (CI) surgery, an increasing number of electrode array designs have been established over the last few decades. In addition, the indication for implantation has been expanded to include patients with residual hearing, especially in low frequencies. Therefore, in addition to years of deafness, age at implantation, scalar position, and neurocognition, other variables influencing the outcome need to be considered (1–4).

The focus of cochlear implant surgeons is on improving outcomes in terms of reducing surgical trauma with the aim of preserving residual hearing. It is well-known that cochlear anatomy varies between patients, requiring the use of different electrode arrays of varying length and design, distinguishing between straight and curved, resulting in individual electrode selection (5, 6). For this reason, the standard preoperative procedure for CI candidates includes either a cone beam computed tomography scan or a computed tomography scan. These imaging techniques enable the surgeon to identify individual variations in temporal bone anatomy and to measure the cochlea, as described in several studies (5, 7, 8). The customization of the electrode array, based on cochlear anatomy, is of increasing interest to manufacturers.

Primary insertion into the scala tympani has been shown to positively influence speech discrimination (1, 4, 9, 10). Perimodiolar electrode arrays, such as the Contour Advance (CA) electrode array developed by Cochlear™, demonstrated dislocation rates of 15.4% and scala vestibuli insertion of 16.1% in the study of Ketterer et al. (11). Previous studies have reported scala tympani dislocation rates ranging from 10 to 38.7%, with the higher rates including cases of scala vestibuli insertions at implantation (19 out of 49 ears implanted with the CA) (12, 13). On the contrary, in 2017, Aschendorff et al. described the slim modiolar electrode array (SMA) as being slim and atraumatic. Ketterer et al. also described very low dislocation rates and a small risk of intracochlear trauma (11, 14). In addition, Aschendorff et al. and Beck et al. described the SMA as a perimodiolar electrode array with a higher risk of tip fold overs, which can be reduced by surgical experience (14, 15). When comparing the electrode arrays of MED-EL, Ketterer et al. found that the risk of scalar dislocation is lower with the short electrode arrays Flex^24^ and Flex^26^ than with the longer electrodes Flex^28^ and Flex^Soft^ (31.5 mm) (6). Furthermore, some studies show that especially short straight arrays have lower dislocation rates than stiffer perimodiolar arrays as the CA (12, 16).

This study aimed to compare audiological residual hearing preservation following CI depending on the implanted electrode array, in particular the difference between straight and perimodiolar electrode array design. We aimed to determine the electrode array design with the least cochlear trauma and thus the best audiological performance regarding residual hearing, and possibly to provide a recommendation for the selection of an electrode array for patients with existing residual hearing.

## Materials and methods

### Study design

We performed a retrospective analysis of adult patients receiving a CI between 2003 and 2021. The study was carried out in the Department of Otorhinolaryngology, Head and Neck Surgery at the Implant Center of the University Hospital Freiburg. Approval from the hospital’s ethics committee according to the Declaration of Helsinki (Washington, 2002) (Number of Ethics Committee approval: 406/19, Amendment number: 230282) was obtained. Patients received Cochlear™ Contour Advance (CI24RECA, CI412/512/612) (CA), Cochlear Slim Straight (422/522/622) (SSA), and Cochlear Slim Modiolar (532/632) (SMA) electrode array, as well as MED-EL Flex^24^ (F24), MED-EL Flex^26^ (F26), MED-EL Flex^28^ (F28), and MED-EL Flex^Soft^ (F31.5) electrodes. The manufacturer was chosen by the patients following individual counseling. The electrode array was selected intraoperatively by the surgeon. This study primarily included all adult patients ≥18 years of age who were implanted with a CI during the years mentioned. Excluded from this study were patients with cochlear anomalies as well as patients with cochlear sclerosis or obliteration identified through preoperatively conducted computed tomography (CT), cone beam CT, or magnetic resonance imaging. One day after CI, we performed postoperative radiological evaluation using a digital volume tomography (New Tom 5G/GXL, Hillus Medical Engineering KG) or a rotational tomography (DynaCT-equipped Axium Artis dTa angiography unit) (Siemens Co., Erlangen, Germany) with a digital flat-panel detector (9, 17). The scans were independently assessed by three physicians with expertise in radiology as well as head and neck surgery.

### Audiological evaluation

Residual hearing was evaluated in a soundproof chamber at frequencies of 250, 500, 1,000, 2000, and 4,000 Hz. From these measured values, the pure tone average for four frequencies (PTA4) was calculated using the frequencies 500, 1,000, 2000, and 4,000 Hz. A total score of <200 for these four frequencies was considered relevant, with the PTA4 threshold defined as an average of <50 dB (200 ÷ 4). If no response was measurable at a tested frequency, a threshold of >120 dB HL was assigned. Residual hearing at low frequencies was defined as a low-frequency pure tone average (PTAlow) of <40 dB HL (summed threshold <80), calculated by averaging the thresholds at 250 and 500 Hz. Both PTA4 and PTA low were measured preoperatively and postoperatively for up to 15.7 years and evaluated for hearing preservation or residual hearing loss using the Kaplan–Meier survival analysis. Postoperative residual hearing loss was defined as an undetectable hearing threshold in the PTA4 cohort or an average hearing threshold of <80 dB HL in the PTA low cohort.

### Statistics

Statistical analysis was performed using Gnu R statistical computation and graphics system (GNU R, Version 3.6.2, Core Team, Vienna, Austria, http://www.R-project.org), extended with the packages nlme (Linear and Nonlinear Mixed Effects Models, Version 3.1, Pinheiro et al., https://CRAN.R-project.org/package=nlme) and ggplot2 (Version 3.3.1, Hadley Wickham, https://ggplot2.tidyverse.org), as well as GraphPad Prism (Version 10, © 2023 GraphPad Software, https://www.graphpad.com/). The calculation of the results was descriptively observed, with the level of statistical significance set at 5.0%.

## Results

### Study cohort

A total of 923 patients who were implanted with a CI between 2003 and 2021 were included in this study. We identified 476 left and 447 right ears. Both straight and perimodiolar electrode arrays from two manufacturers were examined, comprising 724 Cochlear™ and 199 Med-EL electrode arrays. Cochlear™ devices represented the majority, accounting for 78.4% of all implanted devices. The mean age at implantation was 50.5 years.

**Figure 1:**
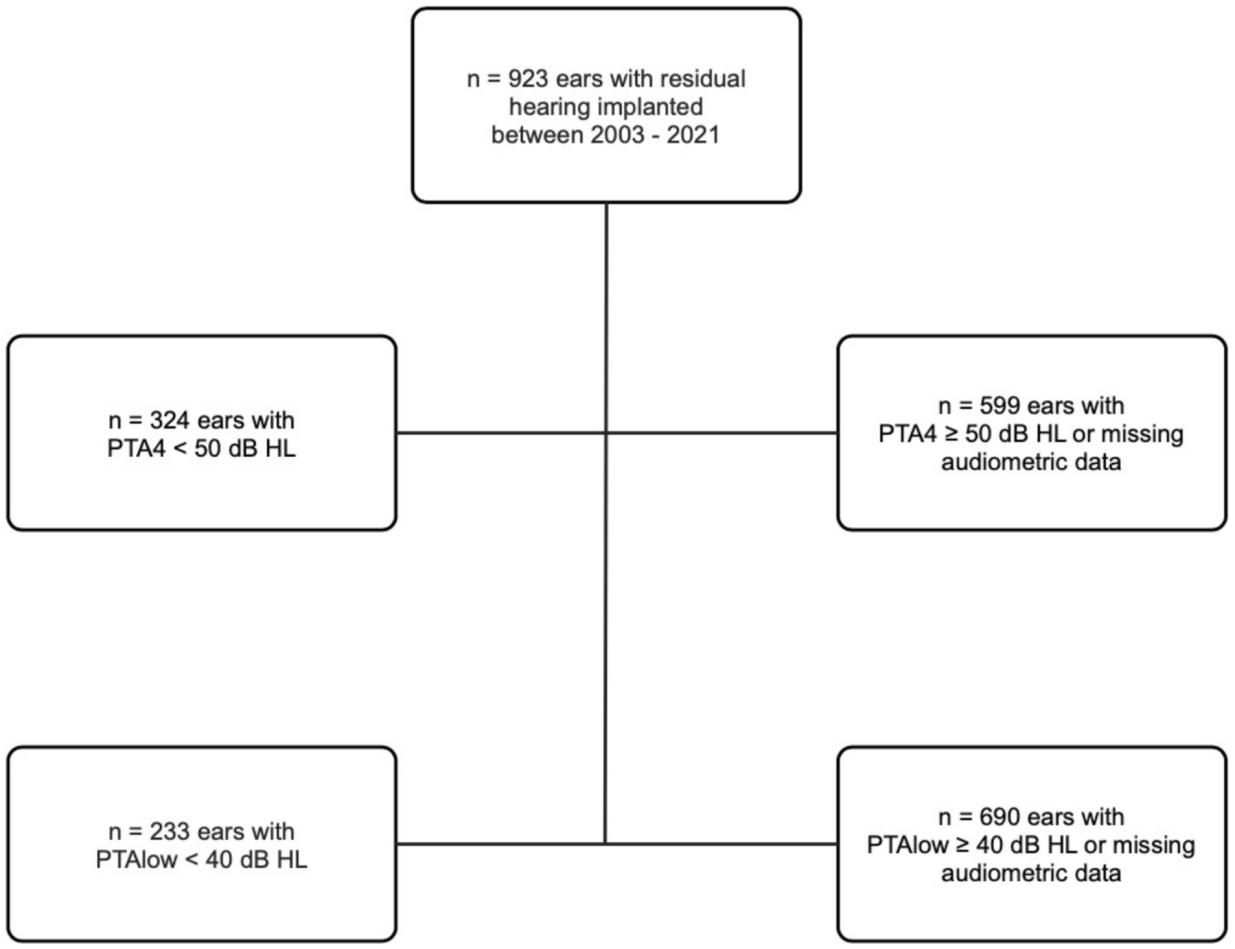
Flowchart of patient cohort (total cohort *n* = 923).

Among the 923 ears, the preoperative PTA4 was below 50 dB in 324 ears, calculated as described by the median of the frequencies shown in Figure 1. Furthermore, PTAlow was evaluated and measured in 233 ears. Table 1 shows the age distribution within the study cohort. Table 2 shows the implant manufacturers and their respective electrode arrays in the total study population.

**Table 1:** Distribution of age of the total study cohort as well as the sub-cohorts with preoperative PTA4 and PTAlow (*n* = 923).

	Age			
	Mean	SD	Max	Min
Total study cohort (*n* = 923)	50.6	17.3	93	16.9
Sub-cohort PTA4 (*n* = 324)	51.1	16.5	86.2	18.2
Sub-cohort PTAlow (*n* = 233)	51.0	15.8	86.2	18.3

**Table 2:** Distribution table of the total study cohort concerning manufacturers and implanted electrode arrays (*n* = 923).

Manufacturer (*n*)	Electrode array	Number (*n*)	Percentage (%)
Cochlear™ (724)	Contour Advance CI 412/512/612 (=CA)	532	57.6
	CI 422/522/622 (=SSA)	169	18.3
	CI 532/632 (=SMA)	23	2.5
Med-El (199)	Flex^24^	28	3.0
	Flex^26^	15	1.6
	Flex^28^	139	15.1
	Flex^Soft^ (31.5)	17	1.8

### Preoperative residual hearing

**Figure 2:**
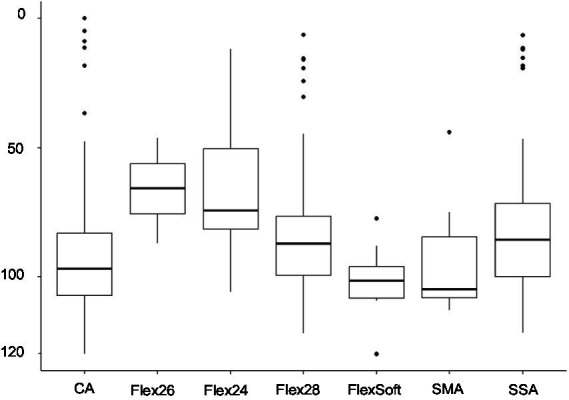
Preoperative PTA4 curves demonstrate the significant influence of the surgeon’s electrode array choice depending on the patient’s choice of manufacturer (*n* = 324) (** Flex^Soft^ vs. Flex^24^ *p* = 0.0085; ***CA vs. SSA *p* = 0.007).

**Figure 3:**
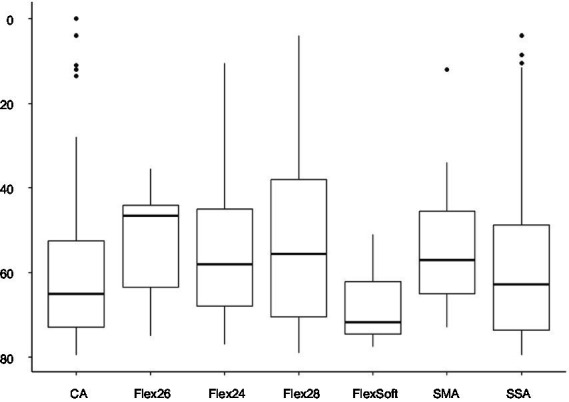
Preoperatively assessed PTAlow (dB) in the sub-cohort of 233 patients shows no statistical difference comparing the selected electrode arrays.

**Figure 4:**
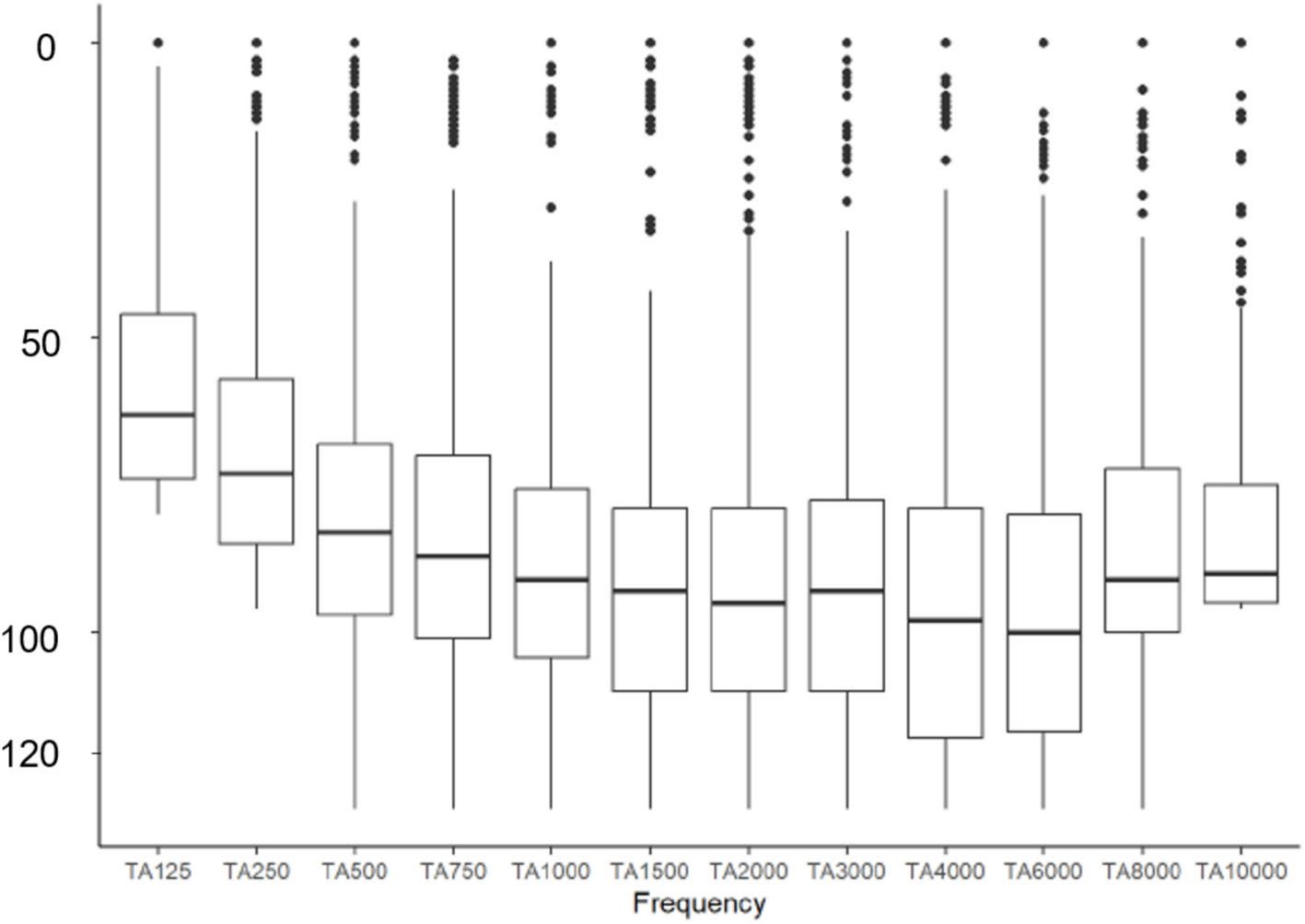
Distribution of hearing loss across frequencies from 125 to 10,000 Hz in the study cohort of 923 ears.

**Figure 5:**
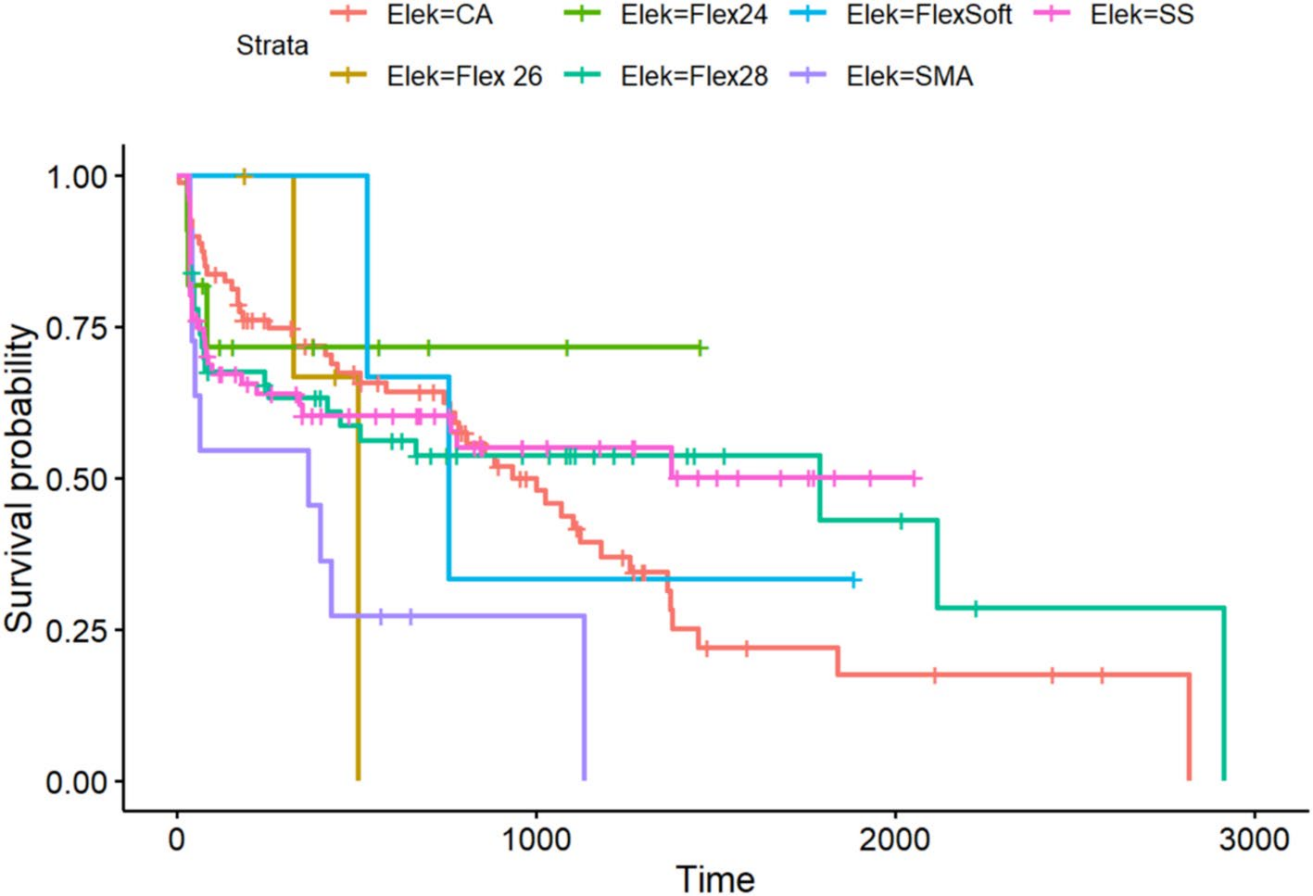
Kaplan–Meier analysis of all included electrode arrays meeting the preoperative inclusion criteria for the PTA4 sub-cohort (*n* = 324).

Among the 324 ears meeting the criteria for preoperatively measurable PTA4, the mean was 92.25 dB (IQR 83–103.25). The preoperatively assessed PTA4 shows significant differences when comparing the various electrode arrays, as demonstrated in Figure 2. For the electrode arrays of MED-EL, the Flex^Soft^ shows a lower PTA4 than the shorter Flex^26^ and Flex^24^, with the difference between Flex^Soft^ and Flex^24^ reaching statistical significance (Flex^Soft^ vs. Flex^24^ *p* = 0.0085). With the electrode array portfolio of Cochlear™, the CA exhibited the lowest preoperatively measured PTA4, with a statistically significant difference compared to SSA (CA vs. SSA *p* = 0.007). Interestingly, when comparing the two included perimodiolar electrode arrays of Cochlear™ CA and SMA, no significant difference was observed (CA vs. SMA *p* = 0.999) (see Figure 2). When comparing preoperative residual hearing at low frequencies within the manufacturer’s portfolio, no statistical significance between the electrode arrays was observed (Figure 3). Differences between the two manufacturers regarding preoperative residual hearing were not analyzed due to the patient’s preoperative choice of the manufacturer. When comparing hearing loss across frequencies, there was an overall increase in hearing loss toward the high frequencies of 6,000 Hz, as shown in Figure 4. For the different electrode arrays, this effect is particularly noticeable with differences between Flex^Soft^, CA, and SMA shown in Figure 5. Figure 4 presents the preoperative hearing loss across all the frequencies ranging from 125 to 10,000 Hz in the study cohort of PTA4, peaking at frequencies from 4,000 to 6,000 Hz.

### Residual hearing preservation

**Figure 6:**
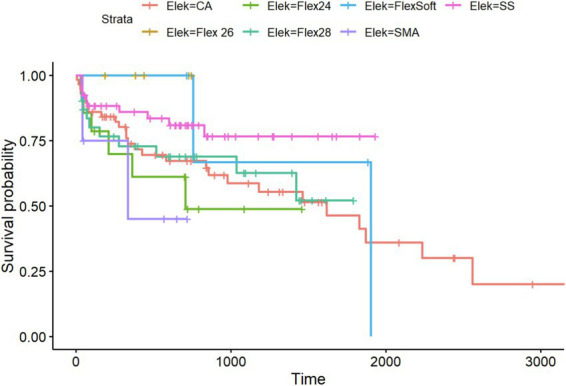
Kaplan–Meier analysis of all included electrode arrays meeting the preoperative inclusion criteria for the PTAlow sub-cohort (*n* = 233).

Regarding residual hearing preservation after surgery with a maximum follow-up of 5,738 days (equivalent to more than 15 years), we also observed differences between the inserted electrode arrays. Overall, in the PTA4 sub-cohort, a significant decrease was observed immediately after implantation in all electrode arrays, with high stability achieved at 5 years, as shown in Figure 6. When comparing residual hearing over time, the short electrodes—SSA from Cochlear™ and Flex^24^ from MED-EL—showed the best results, although statistical significance was lacking. The CA, however, demonstrated the greatest loss of residual hearing over the years (see Figure 5). Among the MED-EL electrode arrays, the differences were less obvious; however, the different number of implanted electrode array groups (*n* = 4 for Flex^26^ in contrast to *n* = 61 for Flex^28^) must be taken into account. When considering the electrode arrays over time after implantation for the PTAlow at the low frequencies of 250 and 500 Hz, the observed effects described were less pronounced, but usable PTAlow was obtained in only 233 ears. In our study cohort, the SSA showed the greatest stability of residual hearing over time, though without statistical significance. Residual hearing preservation was observed across all included electrode arrays (see Figure 6).

## Discussion

Due to further development of electrode arrays with different shapes and lengths as well as improved surgical techniques, CI surgery focuses on reducing intracochlear trauma and preserving residual hearing. As the indication criteria for CI have expanded over the past decade, more patients with existing residual hearing continue to be implanted. This increases the need for an atraumatic electrode array that enables the preservation of the patient’s residual hearing.

Our study shows that the electrode array design significantly influences the preservation of residual hearing, both in low-frequency PTA, i.e., PTAlow, and standard PTA4. The electrode arrays associated with the least cochlear trauma and the most favorable performance in terms of preserving residual hearing were the SSA of Cochlear™ and the Flex^24^ of MED-EL. These straight lateral wall electrode arrays are characterized by a slim design and a short length. Consistent with our findings, previous studies have reported higher rates of hearing preservation with straight and mid-scala electrode arrays (e.g., precurved array from AB) compared to perimodiolar electrode arrays from Cochlear™ (18, 19). Nevertheless, Sweeney et al. included only 16 cases (18). Perkins et al. found a deterioration in low-frequency PTA when comparing patients implanted with precurved electrode arrays from the time of activation to the final follow-up (20). However, they suggested a selection bias, with surgeons favoring straight electrode arrays for patients with higher residual hearing or lower audiometric thresholds (20). This proposal can be validated within the scope of our study as well, as the manufacturer was chosen by the patients, while the specific electrode array was selected intraoperatively by the surgeon. These findings are consistent with another study that proposed that the amount of residual hearing is related to its preservation over the long term (21). The CA accounted for the largest number of included ears in our investigation; therefore, the results regarding residual hearing preservation are robust. The high level of residual hearing loss associated with the CA may be a consequence of traumatic surgical approach, typically cochleostomy, which can trigger fibrotic remodeling and autoimmune processes, leading to scarring. As described in previous studies, this fibro-osseous reaction can cause delayed loss of residual hearing (22, 23). However, it should be noted that fibrotic remodeling after surgery is primarily observed within the first year after implantation, whereas in our study, residual hearing loss was still observed after 1 year (20). Additionally, the composition of the study cohort may have influenced the poorer outcomes associated with the CA, as this CI was preferentially implanted in patients with minimal residual hearing, thus introducing preoperative bias due to the retrospective study design. Despite this, the early implantation of the CA allowed for a longer observation period over the years, suggesting that hearing loss over time may also be due to natural age-related hearing loss.

However, caution should be exercised when interpreting the outcomes regarding residual hearing preservation for the SMA. The SMA ears included in this study were primarily earlier cases in the study cohort, during which surgical procedures were subject to a learning curve, as described by Aschendorff et al. (24). In addition, newly developed electrode arrays are not primarily used in patients for whom the preservation of residual hearing is a secondary goal of CI surgery. Moreover, our dataset encompasses only 23 ears that underwent SMA insertion, with the first implantations following the introduction of this electrode array were not specifically aimed at preserving residual hearing.

Nevertheless, perimodiolar and straight electrode arrays are described to demonstrate different dislocation behaviors and intracochlear trauma risk in multiple studies (1, 5, 11, 13). Variations in the angle between the first and second cochlear turns, as well as diminished lengths typical of underdeveloped cochleae, can exacerbate insertion challenges and elevate the risk of scalar dislocation (25, 26). Another factor influencing the angular and linear insertion depth is the cochlear size, as already described by Ketterer et al. (5). The length of the electrode influences the insertion depth and thus the probability of cochlear trauma. According to our data, short electrode arrays such as the Flex^24^ and the SSA show better outcomes regarding residual hearing. The Flex^26^ did not demonstrate scalar dislocation or radiographically intracochlear trauma, as described by Ketterer et al. (6). However, residual hearing outcomes, as investigated in this study, should be examined with a larger study cohort (*n* = 15). O’Connell reported poorer results in terms of preserving residual hearing with deeper insertion of straight electrode arrays, although this did not reaching statistical significance (27). Other investigations found no difference in angular insertion depth in terms of postoperative shifts in audiometric threshold (20, 28). Erixon et al. included 21 straight electrode arrays and did not find a significant correlation between insertion depth and residual hearing preservation (29). Causon et al. described in their review a significant influence of angular insertion depth on residual hearing (30). Even though most studies described electrode array length as a risk factor for damaging residual hearing (4, 31, 32), some studies did not find such an association (33, 34).

Nevertheless, the preservation of residual hearing depends on many other factors, such as insertion speed, complete opening of the round window membrane, and the use of corticosteroids, as described in multiple previous studies (35–38). Perkins et al. indicated that hearing preservation may reach stability 1 year postoperatively, which could be potentially attributable to insertion trauma and immediate inflammatory responses following surgery (20). However, in our extensive study cohort, this was not confirmed. While Figures 5, 6 demonstrate that the initial decline in residual hearing is most pronounced in the initial days, it is not necessarily stable even 1-year post-implantation, exhibiting a decreasing trend across all electrode arrays examined. Nonetheless, the SSA exhibits the most stable curves over time. Due to the retrospective nature of our study design, we must acknowledge potential bias stemming from incomplete data and the lack of a consistent follow-up interval for determining audiometric thresholds. Additionally, variability between surgeons may serve as a confounding factor. Furthermore, data on the final scalar position as well as the cochlear approach were not collected and analyzed; for example, for round-window insertion, no differences in terms of residual hearing preservation were found when comparing straight and perimodiolar electrodes (39).

## Conclusion

The results presented in this study provide sufficient data to suggest that flexible and slim electrodes are more suitable for residual hearing preservation. In our study, the SSA and Flex^24^, in particular, showed the best results in terms of long-term and stable postoperative residual hearing preservation. However, no statistical difference was observed when compared with other flexible and slim modiolar electrodes, likely due to the number of factors to consider.

In the future, robotic implantation is likely to improve residual hearing preservation outcomes by providing a consistent, atraumatic insertion speed, thereby reducing insertion force as a factor in improving scope results.

## Data Availability

The raw data supporting the conclusions of this article will be made available by the authors, without undue reservation.
